# Female American black bears do not alter space use or movements to reduce infanticide risk

**DOI:** 10.1371/journal.pone.0203651

**Published:** 2018-09-14

**Authors:** D. Cody Norton, Jerrold L. Belant, John G. Bruggink, Dean E. Beyer, Nathan J. Svoboda, Tyler R. Petroelje

**Affiliations:** 1 Department of Biology, Northern Michigan University, Marquette, Michigan, United States of America; 2 Wildlife Division, Michigan Department of Natural Resources, Shingleton, Michigan, United States of America; 3 Camp Fire Program in Wildlife Conservation, State University of New York, College of Environmental Science and Forestry, Syracuse, New York, United States of America; 4 Wildlife Division, Michigan Department of Natural Resources, Marquette, Michigan, United States of America; 5 Division of Wildlife Conservation, Department of Fish and Game, Kodiak, Alaska, United States of America; University of Tasmania, AUSTRALIA

## Abstract

Infanticide occurs in a variety of animal species and infanticide risk has large implications for the evolution of behavior. Further, the sex hypothesis of sexual segregation predicts that for species in which infanticide occurs, females with dependent young will avoid males to reduce risk of sexually-selected infanticide. Infanticide risk-avoidance behavior has been studied primarily in social species, but also occurs in some solitary species. We used generalized linear mixed models to determine if space use and movements of female American black bears (*Ursus americanus*) during the breeding season were consistent with the sex hypothesis of sexual segregation in the Upper Peninsula of Michigan, USA. Space use and movements of female black bears (n = 16) were not consistent with avoidance behavior to reduce sexually-selected infanticide risk. Females with cubs occupied core areas (mean = 4.64 km^2^, standard error [SE] = 1.28) and home ranges (mean = 19.46 km^2^, SE = 5.10) of similar size to females without cubs (core area [mean = 4.11 km^2^, SE = 0.59]; home range [mean = 16.07 km^2^, SE = 2.26]), and those core areas and home ranges were not in areas with lesser relative probability of male use. Additionally, females with cubs did not reduce movements during times of day when male movements were greatest. As female bears do avoid potentially infanticidal males in populations with greater levels of infanticide, female black bears may exhibit variation in avoidance behavior based on the occurrence of infanticide.

## Introduction

Infanticide occurs in a variety of animal species, and infanticide risk has large implications for the evolution of behavior in populations where infanticide is a significant mortality source [1,2]. Sexually-selected infanticide is the killing of dependent offspring by adult conspecifics to increase reproductive opportunities with the opposite sex [2,3]. The sex hypothesis of sexual segregation predicts that risk of infanticide can influence behavior and resource selection in animals, which can produce a despotic distribution as dependent offspring and their respective parents are displaced by potentially infanticidal adult conspecifics [2]. Strategies to reduce infanticide include dispersal, multi-male mating, female selection of a dominant male, male-female associations, intersexual aggression or territoriality, and sexual segregation [1–3]. Sexual segregation occurs when males and females of a species partition resources, which reduces competition and the likelihood of conflict [2,4], including risk of infanticide. Infanticide-avoidance behavior was first studied in social species. For example, female African lions (*Panthera leo*) and dependent young sometimes abandon their prides when a new male takes over to avoid risk of infanticide until the young are weaned [5]. Mountain gorilla (*Gorilla gorilla beringei*) groups may shift territories to avoid infanticide by encroaching males [6]. However, more recently, infanticide risk-avoidance behavior has been observed in solitary species, such as female cougars (*Puma concolor*) with dependent young, which may reduce risk of infanticide by occupying home ranges at lower elevations than males and females without dependent young [7].

Bears (*Ursus* spp.) are solitary species that sometimes commit infanticide [8–11] and exhibit behaviors to reduce this risk. For instance, female brown bears *(U*. *arctos)* select home ranges and habitat types with low male occupancy to reduce risk of sexually-selected infanticide [12]. Risk of sexually-selected infanticide also influenced seasonal range size of female brown bears in Scandinavia, with estrous females occupying larger home ranges during the breeding season than females with cubs [13]. In Sweden, female brown bears with cubs moved shorter distances from den emergence through the breeding season than females without cubs and exhibited differential habitat selection during diurnal periods than males and females without cubs [12,14]. In Alaska, female brown bears also denned earlier, left dens later, and denned at higher elevations than males [15]. Those behavioral changes likely reflected male avoidance strategies in order to reduce the risk of sexually-selected infanticide [15]. Female promiscuity, or multi-male mating, occurs in bear species, and may be employed by females as a counter-strategy to sexually-selected infanticide [17–19]. Female brown bears may mate with up to 8 males in a season [17] and multiple paternity occurs at levels of 14.5–28.0% in litters of 2–3 cubs [18]. Information about the effects of sexually-selected infanticide on black bear behavior and space use is limited despite the fact that in some cases, 45–50% of black bear cub mortality is from infanticide [8,16].

We evaluated whether space use and movements of female American black bears with dependent young were consistent with infanticide-risk-avoidance behavior as expected under the sex hypothesis of sexual segregation. We predicted that during the breeding season: (1) females with cubs would occupy areas with lesser relative probability of male use than core areas and home ranges of females without cubs, (2) females with cubs would occupy smaller core areas and home ranges than females without cubs, and (3) movements of females with cubs would be inversely related to male movements during a given diel period.

## Methods

### Study areas

**Fig 1:**
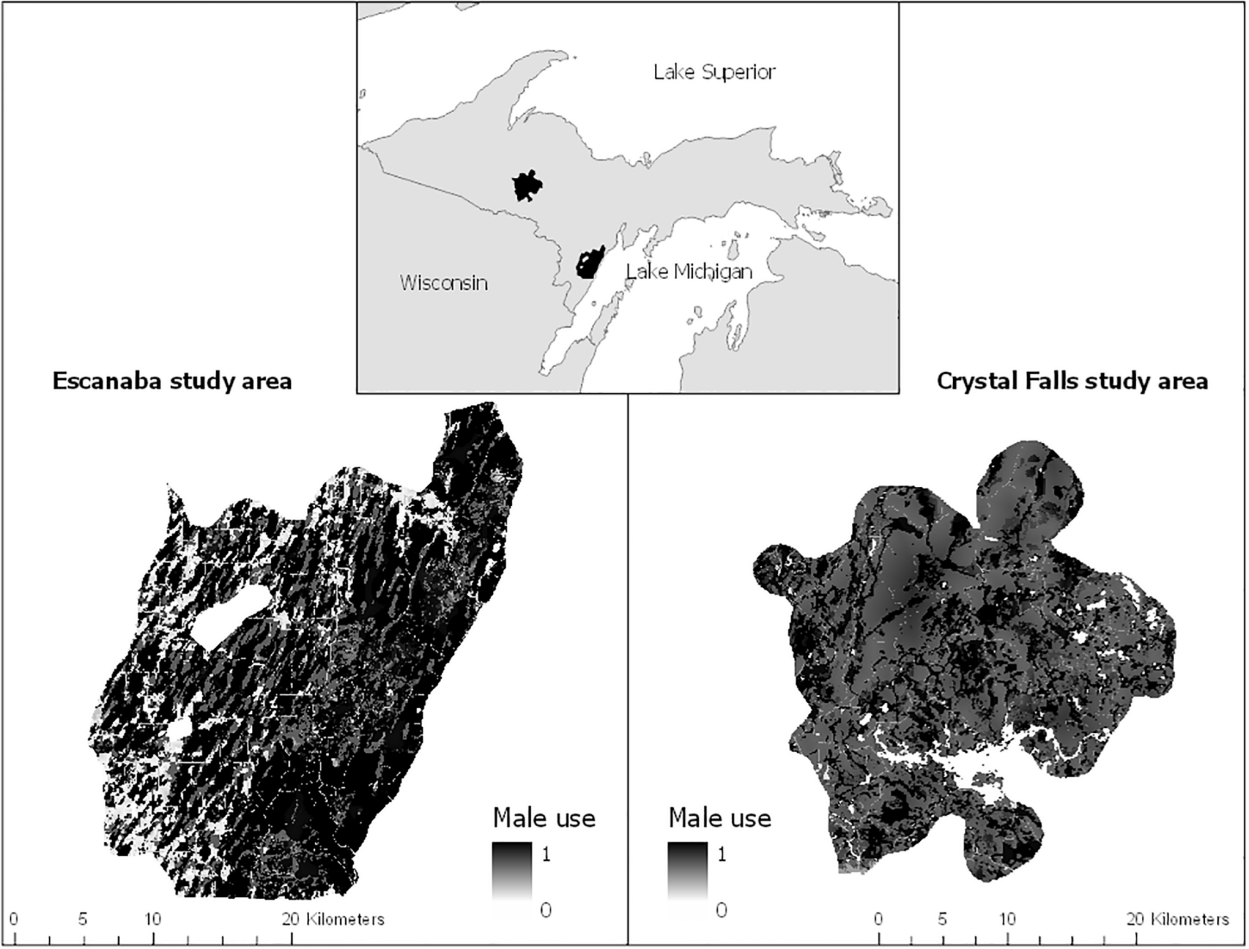
Male use of available area. Relative probability of male American black bear space use in Escanaba (2009–2011, 45.6°N, 87.4°W) and Crystal Falls (2013–2014, 46.3°N, 88.2°W) study areas during the breeding season, Upper Peninsula of Michigan.

We conducted this study in 2 areas within the Upper Peninsula, Michigan, USA. The study area during 2009–2011 (hereafter Escanaba Study Area) included about 850 km^2^ of Delta and Menominee counties (45.6°N, 87.4°E, Fig 1). Land ownership was 72% private and 28% public including the Escanaba River State Forest. Land covers were 52% lowland conifer forests (e.g., black spruce [*Picea mariana*], green ash [*Fraxinus pennsylvanica*], northern white cedar [*Thuja occidentalis*], speckled alder [*Alnus incana*]), 14% deciduous forests (e.g., sugar maple [*Acer saccharum*], quaking aspen [*Populus tremuloides*]), and 14% agriculture (e.g., row crops and pastures). The remaining 20% included upland conifer forests, mixed forests, developed areas, herbaceous wetlands, shrub, and open water [20]. Elevations ranged from 177 to 296 m. From May to September, the average monthly high occurred in July (24.3°C) and the average monthly low occurred in May (3.3°C [21]). Average monthly rainfall was 22.3 cm during May–September 2009–2011. Black bear densities were 14/100km^2^ in 2009, 15/100km^2^ in 2010, and 19/100km^2^ in 2011 (unpublished data).

The study area during 2013–2014 (hereafter Crystal Falls Study Area) included about 1,830 km^2^ of Baraga, Dickinson, Iron, and Marquette counties (46.3°N, 88.2°W, Fig 1). Land ownership was 80% private and 20% public, including the Copper Country and Escanaba River State Forests. Land covers were 36% deciduous forests (e.g., sugar maple, quaking aspen), 31% lowland conifer forests (e.g., black spruce, green ash, northern white cedar, speckled alder), and 12% mixed forests (e.g., sugar maple, eastern hemlock [*Tsuga canadensis*], balsam fir [*Abies balsamea*]). The remaining 21% included shrub, upland conifer forests, open water, developed areas, herbaceous wetlands, and cultivated crops [20]. Elevations ranged from 396 to 579 m. Average monthly high and low temperatures during May–September 2013–2014 were 18.6°C during July and 9.2°C during May, respectively [22]. Average monthly rainfall was 9.4 cm during May–September 2013–2014. Black bear densities were 25/100km^2^ in 2013 and 23/100km^2^ in 2014 (unpublished data).

### Animal capture and telemetry

We captured black bears using barrel traps [23] and modified Aldrich foot snares [24] during May–July 2009–2011 and 2013–2014, which included the bear breeding season in our study areas (1 June–15 July [25]). We immobilized captured bears estimated as ≥2 years old with 4–7 mg/kg Telazol [26]. For each bear, we determined sex and attached a global positioning system (GPS) collar (Model GPS7000MU, Lotek Wireless Inc., Newmarket, Ontario, Canada) that collected and stored locations at 15-minute intervals. We uploaded location data from GPS collars 1–2 times per week from a fixed-wing aircraft. The mean GPS collar fix success rate during the breeding season was 94%. We located bears that retained collars in winter and immobilized them in their dens to replace collars and document number and age (cub or yearling) of offspring. Black bear capture and den checks took place on both private and state lands. Permission to conduct research on private land was granted by the respective landowners. As this was a cooperative study between the Michigan Department of Natural Resources and Mississippi State University, with the university acting as an extension of the state agency, specific permission was not needed to conduct research on state lands. Mississippi State University Institutional Animal Care and Use Committee approved all capturing and handling procedures (protocols 09–004, 12–012).

### Estimating male black bear space use

We estimated the relative male probability use for both study areas to facilitate testing our prediction that females with cubs would occupy core areas and home ranges with lesser probability of male use than females without cubs. We estimated male black bear space use in relation to roads and land cover using bear locations obtained during the breeding season (1 June–15 July [25]) because infanticide events after the breeding season would not increase male opportunities to breed [8]. We included distance to nearest road as roads can influence bear space use and resource selection [27,28]. We included land cover because the composition and abundance of bear foods is strongly related to land cover, and variation in food availability is a primary source of seasonal changes in black bear space use [29,30]. We included the interaction between land cover and distance to nearest road because bears may respond differently to road proximity in different land cover types due to differences in traffic, use for hunting, or other factors [31,32].

We identified roads classified as seasonal secondary or more highly-developed using Michigan Geographic Framework Transportation data [33]. We identified land covers (water, developed/barren, cultivated crops, grass/pasture, deciduous forest, coniferous forest, mixed forest, and woody wetlands) using 2006 National Land Cover Database grid data with 30-m resolution ([20] S1 Table). We combined the original barren land, developed/open, developed/low intensity, developed/medium intensity, and developed/high intensity into a developed/barren category. We combined shrub/scrub, grassland/herbaceous, and pasture/hay and reclassified as grassland/pasture; we combined and reclassified woody wetlands and emergent herbaceous wetlands as wetlands.

We delineated final study areas by creating a 95% and 99% isopleth around all bear locations in the Escanaba and Crystal Falls study areas, respectively, using a fixed kernel density estimator with bandwidth selection [34]. We used different percentage isopleths for each study area to account for differences in the distribution of locations and to ensure relatively contiguous areas. We then generated a grid with 0.4-ha cells which we overlaid on the isopleths and used ArcGIS (Environmental Systems Research Institute, Redlands, California, USA) to determine the number of locations of each male bear in each cell (S1 and S2 Figs). To account for unoccupied areas, we calculated the number of locations in each cell in a respective study area for each bear during the years it was collared. We calculated the distance from the center of each grid cell to the nearest road using Patch Analyst 4.0 for ArcGIS and determined the dominant land cover for each grid cell using the zonal majority routine in ArcGIS [35].

We used generalized linear mixed models (GLMM) with zero-inflated Poisson distributions (log link) to estimate relative probabilistic male black bear use for each study area. Using the number of black bear locations within a given cell as the response variable, we selected the appropriate random effect structure (bear ID, year, or bear ID and year) by identifying the global model with the best fit using Likelihood Ratio Tests. We created a set of candidate models using the glmmTMB package [36] in R 3.5.0 (S3 Table [37]). Candidate models included the best-supported random effect structure, land cover, distance from nearest road, land cover and distance from nearest road, and the interaction between land cover and roads as fixed effects, and number of male locations as the response variable. Zero-inflated models included the intercept, because structural zeros were the result of the analysis design. We used Akaike’s Information Criterion adjusted for small sample size (AIC_C_ [39]) to determine the best-supported model. For each study area, we used the best-supported model to estimate parameters with 95% confidence intervals (CI) and to predict the relative probability of use by males for each cell in the grid (Fig 1).

### Home range and movements

To evaluate relationships between female reproductive status and space use, we used fixed kernel density estimators with bandwidth selection [34] to estimate core areas (50% isopleths) and home ranges (95% isopleths) of females with and without cubs during the breeding season. We then calculated median relative probability of male use in each female core area and home range to evaluate relationships between female reproductive status and male use of female core areas and home ranges. We selected the appropriate random effect structure by including all combinations of random effects (bear ID, year, study area) in global models and identifying the model with the best fit using Likelihood Ratio Tests. We then used GLMM with Gaussian (log link) distribution to compare median relative probabilistic male space use in core areas and home ranges of females with and without cubs. We created a set of candidate models using the lme4 package [38] in R 3.5.0 [37] with the best-supported random effect structure, reproductive status, isopleth type (core area or home range), or reproductive status and isopleth type as fixed effects, and relative male probability of use as the response variable (S4 Table).

We also used generalized linear mixed models (GLMM) with Gaussian (log link) distribution to examine the relationship between reproductive status and the size of core areas and home ranges of females with and without cubs. We selected the appropriate random effect structure by including all combinations of random effect (bear ID, year, study area) in global models and identifying the model with the best fit using Likelihood Ratio Tests. We created a set of candidate models with the best-supported random effect structure, reproductive status (with or without cubs), isopleth type (core area or home range), or reproductive status and isopleth type as fixed effects, and area of core area or home range as the response variable (S5 Table).

To estimate whether females with cubs travel less at times males travel more, we divided each day into morning (0400–0800 hours), day (0801–1929 hours), evening (1930–2330 hours), and night (2331–0359 hours [40]). We calculated distances between consecutive 15-minute locations, summed the distances for each diel period per day, and then calculated the average distance travelled during each diel period. We selected the appropriate random effects structure by including all combinations of random effects (bear ID, year, study area) in global models and identifying the model with the best fit using Likelihood Ratio Tests. We then created a set of candidate models to compare the distance travelled by diel period for each level of sex/reproductive class using GLMM with Gamma (log link) distribution, the best-supported random effect structure, all combinations of sex/reproductive class, diel period, the interaction between sex/reproductive class and diel period, and number of locations as fixed effects, and distance travelled per diel period as the response variable (S6 Table). We used AIC_C_ to evaluate support among candidate models for all female home range and movement analyses. We considered models with ΔAIC_C_ ≤ 2 to have equivalent support to the best-supported model [39]. However, if a competing model had a ΔAIC_C_ ≤ 2 of the top model, and the parameters in one model were a subset of the parameters in a competing model, we selected the simpler model in order to avoid inclusion of uninformative parameters [41].

## Results

We captured and placed GPS collars on 18 male and 8 female black bears in the Escanaba study area and 8 males and 8 females in the Crystal Falls study area. Overall, we used 29 male, 12 female with cubs, and 10 female without cubs bear-season combinations for analyses, which provided 151,642 locations. During breeding season, the mean number of locations per individual was 2,346 (SD = 1,372) for males, which equates to about 24 days and 3,252 (SD = 1,465) for females, which equates to about 34 days.

**Fig 2:**
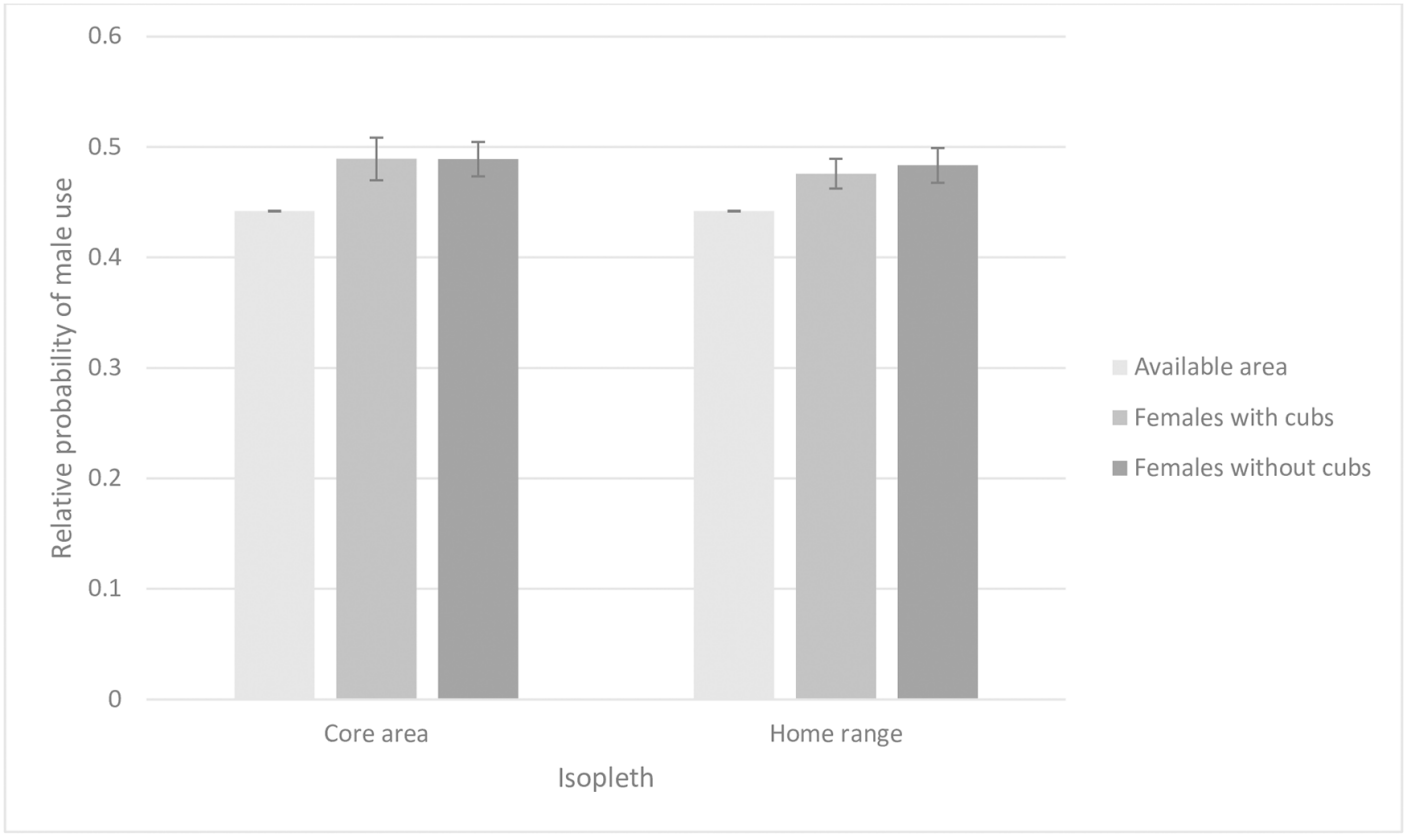
Male use in female core areas and home ranges. Relative probability of male American black bear use in core areas (50% kernel) and home ranges (95% kernel) of females with and without cubs, Upper Peninsula of Michigan, 2009–2011 and 2013–2014. Error bars represent 1 standard error.

The best-supported models for estimating relative probability of male use in both study areas included land cover, distance from nearest road, and their interaction as fixed effects (S2 Table). The model with the lowest AIC_C_ for estimating median relative probability of male use in female core areas and home ranges included isopleth type (core area or home range) as a fixed effect. However, we found similar support for the null model (ΔAICc = 0.43) and the model that contained isopleth type and reproductive status (ΔAICc = 2.00). Because parameters in the null model were a subset of the competing models, we considered the null model best-supported (S4 Table). Because reproductive status was not included in the best-supported model, we concluded that females with cubs occupied core areas and home ranges with similar relative probability of male use as females without cubs (Fig 2, Table 1).

**Table 1:** Size and male use of female core areas and home ranges.

Study area	Reproductive status	Size				Relative probability of male use			
		Core area		Home range		Core area		Home range	
		Mean	SD	Mean	SD	Mean	SD	Mean	SD
Escanaba	Females with cubs	6.36	6.02	26.07	23.67	0.44	0.02	0.43	0.03
	Females without cubs	5.55	1.78	22.03	5.13	0.45	0.04	0.44	0.03
Crystal Falls	Females with cubs	2.92	0.67	12.85	4.68	0.54	0.06	0.52	0.02
	Females without cubs	2.97	0.64	11.32	2.99	0.52	0.02	0.52	0.02

Size (km^2^) of female American black bear core areas and home ranges with and without cubs and relative probability of male use, Upper Peninsula of Michigan, 2009–2011 and 2013–2014.

**Fig 3:**
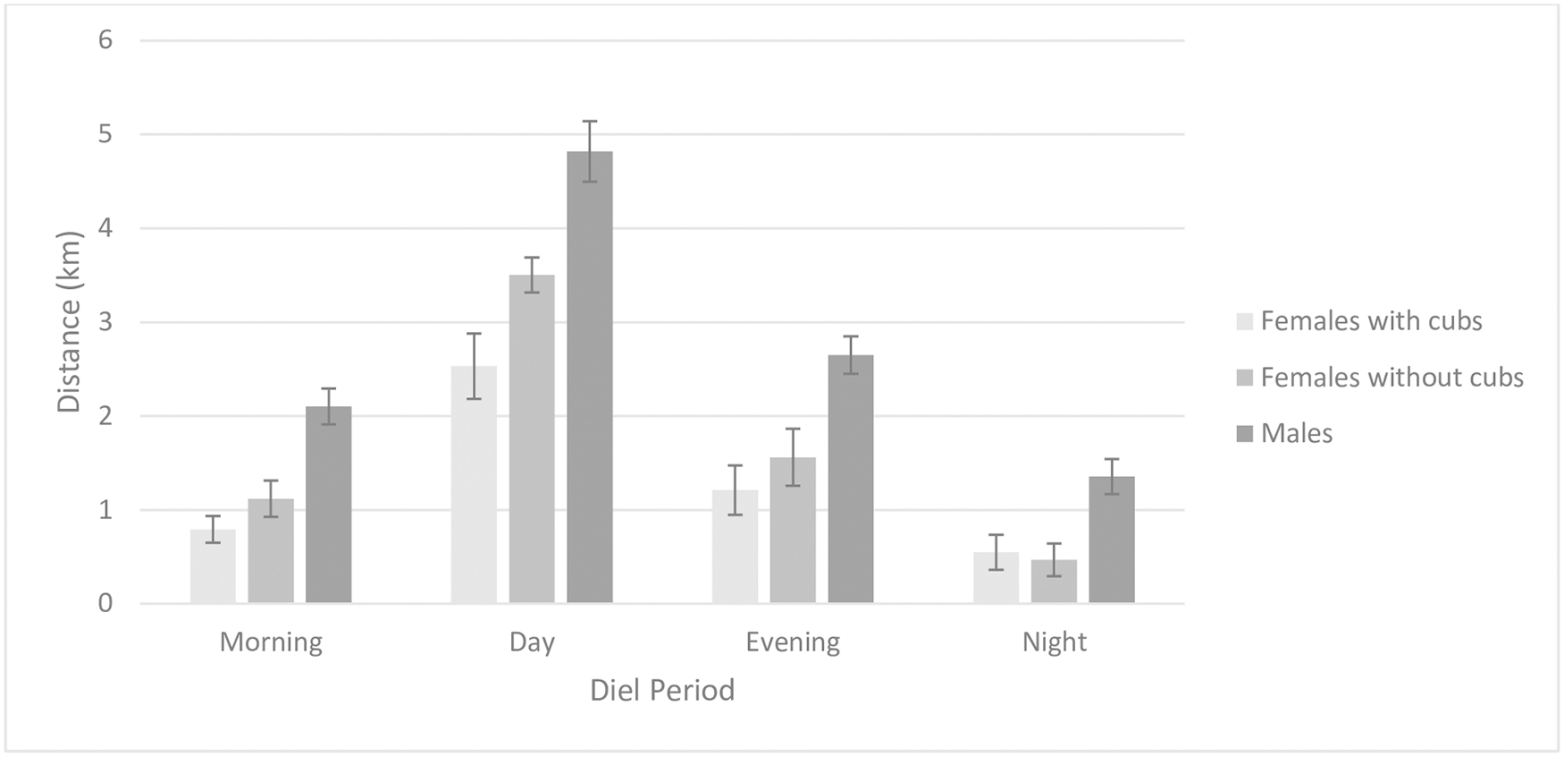
Distance travelled during diel periods. Distance travelled during diel periods (morning [0400–0800 hours], day [0801–1929 hours], evening [1930–2330 hours] and night [2331–0359 hours]) by male and female American black bears with and without cubs, Upper Peninsula of Michigan, 2009–2011 and 2013–2014. Error bars represent 1 standard error.

The best-supported model for examining the relationship between reproductive status and the size of core areas and home ranges of females included isopleth type (core area or home range) as a fixed effect. We found similar support (ΔAICc = 1.96) for the model that included isopleth type and reproductive status but because parameters in the top model were a subset of parameters in the competing model, we only considered the model containing isopleth type as best-supported. (S5 Table). Because reproductive status was not included in the best-supported model, we concluded that females with cubs occupied core areas (mean = 4.64 km^2^, SE = 1.28) and home ranges (mean = 19.46 km^2^, SE = 5.10) of similar size to females without cubs (core area mean = 4.11 km^2^, SE = 0.59; home range mean = 16.07 km^2^, SE = 2.26). The best-supported model for estimating distance travelled per diel period included diel period, number of locations, and sex/reproductive status as fixed effects. We found less support (ΔAICc ≥ 4.88) for models that included the interaction between sex/reproductive status and diel period (Table 2, Fig 3, S6 Table).

**Table 2:** Distance travelled per day and diel period.

Study area	Sex/reproductive status	24-hour period		Morning		Day		Evening		Night	
		Mean	SD	Mean	SD	Mean	SD	Mean	SD	Mean	SD
Escanaba	Males	10.41	3.93	1.71	0.89	4.55	1.85	2.63	1.16	1.51	1.10
	Females with cubs	5.40	3.59	0.86	0.68	2.19	1.20	1.44	1.12	0.90	0.79
	Females without cubs	7.54	2.57	0.93	0.61	3.55	0.44	2.29	0.91	0.77	0.72
Crystal Falls	Males	12.31	1.49	3.13	0.52	5.53	1.25	2.70	0.90	0.95	0.61
	Females with cubs	4.78	2.07	0.72	0.24	2.87	1.21	0.98	0.67	0.20	0.05
	Females without cubs	5.94	1.17	1.27	0.58	3.46	0.69	0.98	0.28	0.23	0.03

Distance (km) travelled per 24-hour period and per diel period (morning [0400–0800 hours], day [0801–1929 hours], evening [1930–2330 hours] and night [2331–0359 hours]) by male and female American black bears with and without cubs, Upper Peninsula of Michigan, 2009–2011 and 2013–2014.

## Discussion

Observed space use and movements of female black bears were not consistent with the sex hypothesis of sexual segregation. Contrary to our predictions, females with cubs did not avoid males spatially or temporally during the breeding season. Rather, irrespective of reproductive status, females used areas with similar relative probability of male use, occupied core areas and home ranges of similar size, and traveled similar distances including at times of day when males traveled more. An alternative to the sex hypothesis of sexual segregation predicts that females with cubs will have decreased space use and movements during spring due to limited mobility of cubs [42–44]. However, our results do not support this alternative hypothesis because we did not detect differences in space use and movements between females with and without cubs (e.g., [45]).

The lack of avoidance behavior by female black bears as demonstrated by space use and movements suggests the risk of sexually-selected infanticide is not great enough to cause strong behavioral changes in these populations. Therefore, sexually-selected infanticide may not occur, or may occur at lesser levels in our black bear population than has been observed in other bear species. Other species predicted to exhibit sexually-selected infanticide also have not demonstrated these behaviors. For example, male degus (*Octodon degus*) did not exhibit infanticidal behavior, even though it should be beneficial due to their social structure and breeding system [46]. Although infanticide is a large mortality source in some black bear populations, infanticide also may vary across populations within species, such as in some North American bear populations in which infanticide appears to be explained by nutritional gain or reduced competition rather than increased breeding opportunities [8,16,47–49].

The black bear populations we studied are hunted and we assume experience relatively high male turnover, as annual apparent hunting mortality was 25% to 44%, with males composing 61% of the harvest (unpublished data). There are two competing hypotheses to predict the impacts of the harvest of males of a species that exhibits sexually-selected infanticide. The immigrant male hypothesis predicts the harvest of males will increase offspring mortality as immigrant males move in and resident males spatially reorganize to replace harvested resident males and kill offspring in the new area to breed with females sooner [7,11,47,50]. Conversely, the mate-recognition hypothesis predicts that the harvest of males can actually decrease offspring mortality in populations, since infanticide risk is likely to decrease as the number of males in the population decreases [51,52]. The mate-recognition hypothesis suggests that males can recognize females they have bred with and take advantage of any opportunity to commit sexually-selected infanticide, whether they are new immigrants in an area or already established residents [51,52]. Our results do not support the immigrant male hypothesis, as females did not reduce core area and home range sizes, avoid areas with high relative probability male use, or travel less distance per day or at times of day males travel more to decrease sexually-selected infanticide risk in these hunted populations. However, it is possible that females with cubs in our study only avoid males with whom they did not mate during the previous breeding season, so the mate-recognition hypothesis may explain the lack of spatial and temporal sexual segregation (e.g., African lions [53]).

Though the causes of variation in rates of infanticide among black bear populations remain unknown, infanticide can be a major source of cub mortality in some populations, representing up to 50% of 46–48% overall annual mortalities [8,16]. Although infanticide has been documented in the Upper Peninsula of Michigan [54], it does not appear to be an important source of mortality here or in surrounding areas. Cub mortality in Minnesota was 25% with no infanticide detected [55]. Cub mortality in the northern Lower Peninsula of Michigan also was 25%, but cause-specific mortality was not estimated [56]. Similarly, annual cub mortality in our study areas averaged 22% (unpublished data), which is less than populations in Arizona (48%) and Florida (46%) that have relatively high rates of infanticide [8,16].

For an infanticidal male to increase breeding opportunities, he would have to kill a female’s entire litter. Brown bear populations in Alaska with lesser rates of complete litter loss (14–26% [53]) experienced low infanticide rates and greater levels of harvest, while brown bear populations in Alaska and Sweden with greater rates of complete litter loss (35–59%) had greater infanticide rates and lesser harvest levels [10,53]. Though cause-specific mortality of cubs in our study was not investigated, we observed only one case of complete litter loss in 11 litters in our study areas (unpublished data), which also suggests sexually-selected infanticide was not an important mortality source.

A limitation of our study is that we classified females as being with or without cubs during a given breeding season based on whether or not they had cubs in the previous den check and/or yearlings in the following den check. It is possible that a female could have lost her litter before a breeding season and been incorrectly classified as a female with cubs. However, our low cub mortality rate and low incidence of complete litter loss suggest this is uncommon and should have had minimal impacts on our results.

Infanticide-avoidance behavior and the sex hypothesis of sexual segregation originated from studies of social species [2,3,5,6]. However, sexually-selected infanticide can be an important source of mortality in solitary species [4,7,8,16]. Studies that document cause-specific mortality of offspring at varying rates of harvest and population densities are needed to better understand the role of infanticide in black bears. Additionally, quantifying the effects of proximity and relatedness between male and female black bears during the breeding season on infanticide could support or refute the immigrant male or mate-recognition hypotheses. Further research is needed to determine the pervasiveness of behavior consistent with the sex hypothesis of sexual segregation in solitary mammal species, and for other taxa (e.g., black rock skinks [*Egernia saxatilis*] [57]).

## Supporting information

S1 Fig Distribution of male locations in Escanaba. The distribution of the number of male black bear locations per grid cell within the Escanaba study area, Upper Peninsula of Michigan, 2009–2011.
(DOCX)

S2 Fig Distribution of male locations in Crystal Falls. The distribution of the number of male black bear locations per grid cell within the Crystal Falls study area, Upper Peninsula of Michigan, 2013–2014.
(DOCX)

S1 Table Land covers used to assess space use. Land covers used to assess American black bear space use in Michigan, 2009–2011 and 2012–2013. Land covers were reclassified from 2006 and 2011 National Land Cover Database.
(DOCX)

S2 Table Candidate models used to estimate male space use. Candidate models used to estimate relative male American black bear space use in Michigan, 2009–2011 (Escanaba study area) and 2012–2013 (Crystal Falls study area).
(DOCX)

S3 Table Male habitat selection parameter estimates. Conditional model habitat selection parameter estimates for male American black bears during the breeding season (1 June–15 July) in Michigan, 2009–2011 and 2013–2014. Zero-inflation models included intercept (Escanaba [estimate = 5.006, SE = 0.008], Crystal Falls [estimate = 4.529, SE = 0.011]). The reference category is water.
(DOCX)

S4 Table Candidate models for male use in female core areas and home ranges. Candidate models used to compare relative male American black bear space use in female core areas and home ranges in Michigan, 2009–2011 and 2012–2013. Fixed effects included reproductive status (with or without cubs) and isopleth type (core area or home range).
(DOCX)

S5 Table Candidate models for female core area and home range size. Candidate models used to compare core area and home range size of female American black bears with and without cubs in Michigan, 2009–2011 and 2012–2013. Fixed effects included reproductive status (with or without cubs) and isopleth type (core area or home range).
(DOCX)

S6 Table Candidate models for distance travelled during diel periods. Candidate models used to compare distance travelled by American black bears during diel periods in Michigan, 2009–2011 and 2012–2013. Fixed effects included sex/reproductive status (male, female with cubs, female without cubs), diel period (morning, day, evening, night), locations (number of locations), and the interaction between sex/reproductive status and diel period.
(DOCX)
