# Supplementary figures and images for: Female American black bears do not alter space use or movements to reduce infanticide risk
Source: PLoS One. 2018 Sep 14;13(9):e0203651. doi: 10.1371/journal.pone.0203651 (PMC6138387; doi:10.1371/journal.pone.0203651)

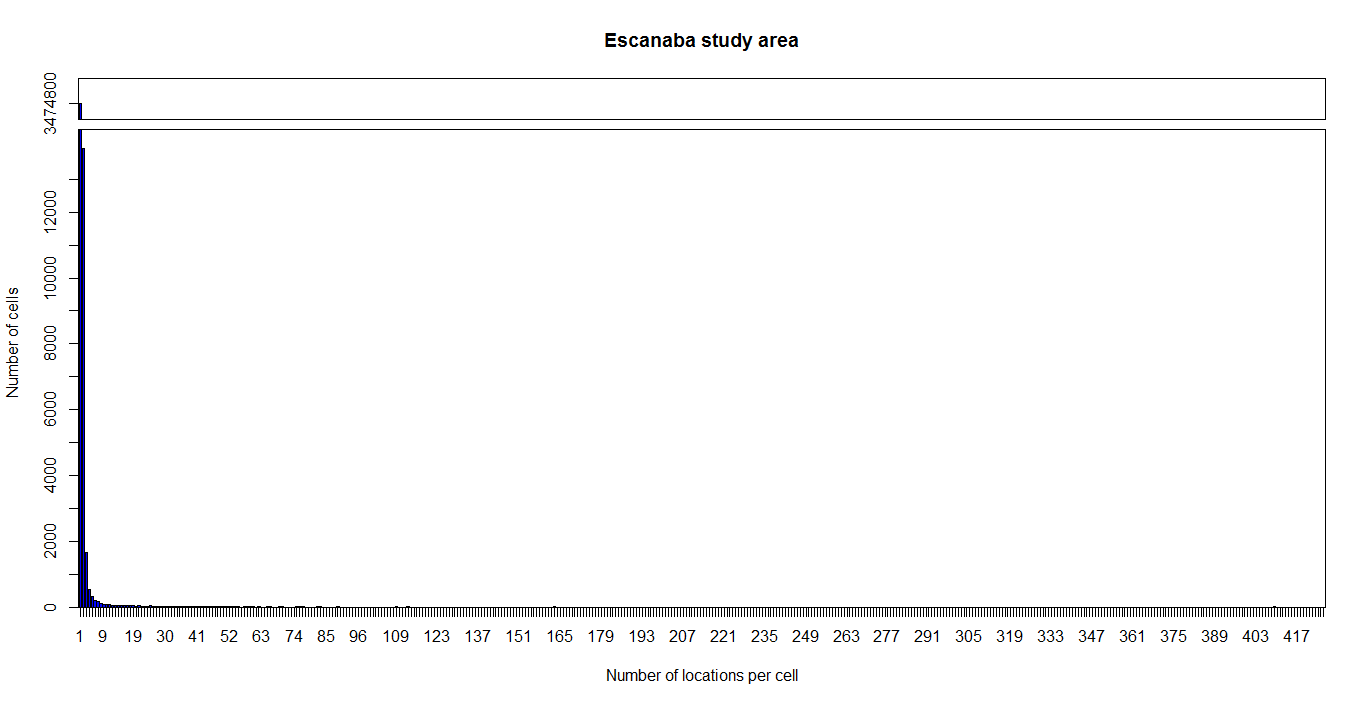

Supplement: S1 Fig — The distribution of the number of male black bear locations per grid cell within the Escanaba study area, Upper Peninsula of Michigan, 2009–2011. (DOCX) [file pone.0203651.s001.docx]

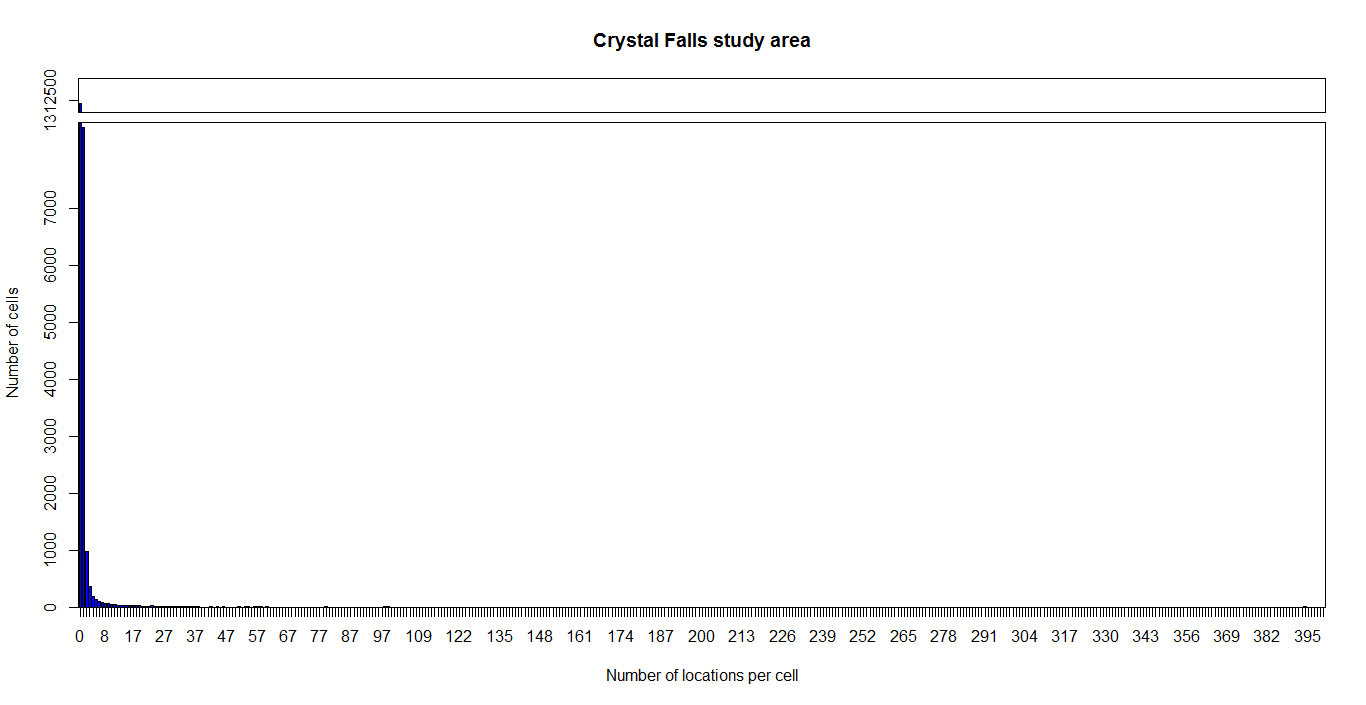

Supplement: S2 Fig — The distribution of the number of male black bear locations per grid cell within the Crystal Falls study area, Upper Peninsula of Michigan, 2013–2014. (DOCX) [file pone.0203651.s002.docx]
