# Supplementary material for: Female American black bears do not alter space use or movements to reduce infanticide risk
Source: PLoS One. 2018 Sep 14;13(9):e0203651. doi: 10.1371/journal.pone.0203651 (PMC6138387; doi:10.1371/journal.pone.0203651)
Supplement: S1 Table — Land covers used to assess American black bear space use in Michigan, 2009–2011 and 2012–2013. Land covers were reclassified from 2006 and 2011 National Land Cover Database. (DOCX) [file pone.0203651.s003.docx]

| **Land cover** | **Definition** |
| --- | --- |
| Water | Areas of open water, generally with <25% cover or vegetation or soil |
| Developed/barren | Areas with anthropogenic materials or structures, or <15% vegetative cover |
| Deciduous forest | Forest with >75% deciduous trees that are >5 m tall and >20 % vegetation cover |
| Coniferous forest | Forest with >75% coniferous trees that are >5 m tall and >20% vegetation cover |
| Mixed forest | Forest with a mix of deciduous and coniferous trees that individually comprise <75% tree cover |
| Grass/pasture | Vegetation >80% graminoid or herbaceous, or trees or shrubs <5 m tall, or grasses or legumes for livestock grazing or production of seed or hay crop |
| Cultivated crops | Area with >20% used for row crop production, including orchards and land actively tilled |
| Wetlands | Areas periodically saturated with water and >20% forest or shrub cover |
