# Supplementary material for: Female American black bears do not alter space use or movements to reduce infanticide risk
Source: PLoS One. 2018 Sep 14;13(9):e0203651. doi: 10.1371/journal.pone.0203651 (PMC6138387; doi:10.1371/journal.pone.0203651)
Supplement: S2 Table — Candidate models used to estimate relative male American black bear space use in Michigan, 2009–2011 (Escanaba study area) and 2012–2013 (Crystal Falls study area). (DOCX) [file pone.0203651.s004.docx]

|  | Escanaba | | | | |
| --- | --- | --- | --- | --- | --- |
| Model | AIC_C_ | ΔAIC_C_ | *w* | log likelihood | *K* |
| roads + land cover + roads * landcover | 334167.1 | 0.0 | 1.0 | -167065.5 | 17 |
| land cover | 334625.8 | 458.7 | 0.0 | -167302.9 | 9 |
| roads + land cover | 334627.3 | 460.2 | 0.0 | -167302.6 | 10 |
| roads | 337746.8 | 3579.7 | 0.0 | -168869.4 | 3 |
| null | 337768.6 | 3601.5 | 0.0 | -168881.3 | 2 |

|  | Crystal Falls | | | | |
| --- | --- | --- | --- | --- | --- |
| Model | AIC_C_ | ΔAIC_C_ | *w* | log likelihood | *K* |
| roads + land cover + roads * landcover | 182747.8 | 0.0 | 1.0 | -91355.9 | 17 |
| roads + land cover | 182778.3 | 30.5 | 0.0 | -91378.1 | 10 |
| land cover | 182829.4 | 81.6 | 0.0 | -91404.7 | 9 |
| roads | 184219.5 | 1471.7 | 0.0 | -92105.8 | 3 |
| null | 184287.5 | 1539.7 | 0.0 | -92140.7 | 2 |
