# Supplementary material for: Female American black bears do not alter space use or movements to reduce infanticide risk
Source: PLoS One. 2018 Sep 14;13(9):e0203651. doi: 10.1371/journal.pone.0203651 (PMC6138387; doi:10.1371/journal.pone.0203651)
Supplement: S3 Table — Conditional model habitat selection parameter estimates for male American black bears during the breeding season (1 June–15 July) in Michigan, 2009–2011 and 2013–2014. Zero-inflation models included intercept (Escanaba [estimate = 5.006, SE = 0.008], Crystal Falls [estimate = 4.529, SE = 0.011]). The reference category is water. (DOCX) [file pone.0203651.s005.docx]

|  | Escanaba | | | Crystal Falls | | |
| --- | --- | --- | --- | --- | --- | --- |
| Model term | Parameter estimate | 95% Confidence limit | | Parameter estimate | 95% Confidence limit | |
|  |  | Lower | Upper |  | Lower | Upper |
| Intercept | -1.788 | -0.905 | -2.672 | -3.052 | -2.663 | -3.441 |
| Coniferous forest | 2.704 | 3.522 | 1.886 | 3.578 | 3.935 | 3.221 |
| Cultivated crops | 0.068 | 0.969 | -0.833 | 3.593 | 4.301 | 2.885 |
| Deciduous forest | 2.340 | 3.157 | 1.523 | 3.453 | 3.805 | 3.101 |
| Developed/barren | 1.474 | 2.303 | 0.646 | 2.967 | 3.335 | 2.600 |
| Grass/pasture | 1.183 | 2.003 | 0.363 | 3.674 | 4.027 | 3.320 |
| Mixed Forest | 2.386 | 3.204 | 1.567 | 3.450 | 3.803 | 3.096 |
| Wetlands | 2.747 | 3.564 | 1.931 | 3.833 | 4.185 | 3.480 |
| Roads | 0.380 | 1.780 | -1.019 | -0.214 | 0.185 | -0.612 |
| Coniferous forest * Roads | -0.409 | 0.991 | -1.809 | 0.252 | 0.655 | -0.151 |
| Cultivated Crops * Roads | -0.137 | 1.351 | -1.625 | 2.205 | 3.282 | 1.128 |
| Deciduous forest * Roads | -0.390 | 1.009 | -1.790 | 0.267 | 0.666 | -0.133 |
| Developed/barren * Roads | -0.291 | 1.113 | -1.696 | 0.270 | 0.677 | -0.138 |
| Grass/pasture * Roads | 0.279 | 1.680 | -1.121 | 0.350 | 0.750 | -0.050 |
| Mixed forest * Roads | -0.488 | 0.912 | -1.889 | 0.268 | 0.669 | -0.132 |
| Wetlands * Roads | -0.391 | 1.009 | -1.790 | 0.218 | 0.617 | -0.182 |
