# Supplementary material for: Female American black bears do not alter space use or movements to reduce infanticide risk
Source: PLoS One. 2018 Sep 14;13(9):e0203651. doi: 10.1371/journal.pone.0203651 (PMC6138387; doi:10.1371/journal.pone.0203651)
Supplement: S5 Table — Candidate models used to compare core area and home range size of female American black bears with and without cubs in Michigan, 2009–2011 and 2012–2013. Fixed effects included reproductive status (with or without cubs) and isopleth type (core area or home range). (DOCX) [file pone.0203651.s007.docx]

| Model | AIC_C_ | ΔAIC_C_ | *w* | log likelihood | *K* |
| --- | --- | --- | --- | --- | --- |
| isopleth type | 732.42 | 0.00 | 0.73 | -361.67 | 4 |
| reproductive status + isopleth type | 734.38 | 1.96 | 0.27 | -361.36 | 5 |
| null | 750.69 | 18.27 | 0.00 | -372.03 | 3 |
| reproductive status | 752.88 | 20.46 | 0.00 | -371.90 | 4 |
