# Supplementary material for: Female American black bears do not alter space use or movements to reduce infanticide risk
Source: PLoS One. 2018 Sep 14;13(9):e0203651. doi: 10.1371/journal.pone.0203651 (PMC6138387; doi:10.1371/journal.pone.0203651)
Supplement: S6 Table — Candidate models used to compare distance travelled by American black bears during diel periods in Michigan, 2009–2011 and 2012–2013. Fixed effects included sex/reproductive status (male, female with cubs, female without cubs), diel period (morning, day, evening, night), locations (number of locations), and the interaction between sex/reproductive status and diel period. (DOCX) [file pone.0203651.s008.docx]

| Model | AIC_C_ | ΔAIC_C_ | *w* | log likelihood | *K* |
| --- | --- | --- | --- | --- | --- |
| diel period + locations + sex/reproductive status | 3295.76 | 0.00 | 0.90 | -1637.30 | 10 |
| diel period + locations + sex/reproductive status + sex/reproductive status * diel period | 3300.64 | 4.88 | 0.08 | -1094.15 | 16 |
| diel period + sex/reproductive status | 3303.22 | 7.46 | 0.02 | 1642.14 | 9 |
| diel period + sex/reproductive status + sex/reproductive status * diel period | 3307.36 | 11.59 | 0.00 | -1632.83 | 15 |
| diel period + locations | 3320.75 | 24.99 | 0.00 | -1652.00 | 8 |
| diel period | 3326.72 | 30.95 | 0.00 | -1656.07 | 7 |
| locations + sex/reproductive status | 3375.36 | 79.60 | 0.00 | -1680.39 | 7 |
| locations | 3395.51 | 99.74 | 0.00 | -1692.60 | 5 |
| sex/reproductive status | 3450.81 | 155.04 | 0.00 | -1719.19 | 6 |
| null | 3468.44 | 172.68 | 0.00 | -1730.12 | 4 |
